# A systematic review of primary large cell neuroendocrine carcinoma of the prostate

**DOI:** 10.3389/fonc.2024.1341794

**Published:** 2024-03-07

**Authors:** Ngan Nguyen, Ronald Dean Franz, Omar Mohammed, Richard Huynh, Christine Kim Son, Rida Nusrat Khan, Bilawal Ahmed

**Affiliations:** ^1^ Hematology and Medical Oncology, The Oncology Institute of Hope and Innovation, Riverside, CA, United States; ^2^ College of Medicine, The University of Tennessee Health Science Center (UTHSC), Memphis, TN, United States; ^3^ Department of Hematology and Oncology, The University of Tennessee Health Science Center (UTHSC), Memphis, TN, United States; ^4^ Department of Internal Medicine, The University of Tennessee Health Science Center (UTHSC), Memphis, TN, United States

**Keywords:** genitourinary cancer, prostate cancer, neuroendocrine cancer, systematic review, prostate specific antigen, large cell neuroendocrine carcinoma, adenocarcinoma

## Abstract

**Background:**

Large cell neuroendocrine carcinoma (LCNEC) is a rare subtype of prostate cancer. The pathogenesis, clinical manifestation, treatment options, and prognosis are uncertain and underreported.

**Materials and methods:**

A systematic search was conducted in April 2022 through PubMed, Embase, and Cochrane. We reviewed cases of LCNEC developed either from *de novo* or transformation from prostate adenocarcinoma and summarized the relevant pathophysiological course, treatment options, and outcomes.

**Results:**

A total of 25 patients with a mean age of 70.4 (range 43 87 years old) from 18 studies were included in this review. 13 patients were diagnosed with *de novo* LCNEC of the prostate. 12 patients were from the transformation of adenocarcinoma post-hormonal therapy treatment. Upon initial diagnosis, patients diagnosed with *de novo* prostatic LCNEC had a mean serum PSA value of 24.6 ng/ml (range: 0.09-170 ng/ml, median 5.5 ng/ml), while transformation cases were significantly lower at 3.3 ng/ml (range: 0-9.3 ng/ml, median 0.05 ng/ml). The pattern of metastasis closely resembles prostate adenocarcinoma. Six out of twenty-three cases displayed brain metastasis matching the correlation between neuroendocrine tumors and brain metastasis. Three notable paraneoplastic syndromes included Cushings syndrome, dermatomyositis, and polycythemia. Most patients with advanced metastatic disease received conventional platinum-based chemotherapy with a mean survival of 5 months. There was one exception in the transformation cohort with a somatic BRCA2 mutation who was treated with a combination of M6620 and platinum-based chemotherapy with an impressive PFS of 20 months. Patients with pure LCNEC phenotype have worse survival outcomes when compared to those with mixed LCNEC and adenocarcinoma phenotypes. It is unclear whether there is a survival benefit to administering ADT in pure pathologies.

**Conclusion:**

LCNEC of the prostate is a rare disease that can occur *de novo* or transformation from prostatic adenocarcinoma. Most patients present at an advanced stage with poor prognosis and are treated with conventional chemotherapy regimens. Patients who had better outcomes were those who were diagnosed at an early stage and received treatment with surgery or radiation and androgen deprivation therapy (ADT). There was one case with an exceptional outcome that included a treatment regimen of M6620 and chemotherapy.

## Background

Large Cell Neuroendocrine Carcinoma (LCNEC) is a subtype of neuroendocrine prostate cancer. Similar to other neuroendocrine histologies such as adenocarcinoma with neuroendocrine differentiation and small cell carcinoma of the prostate, patients with LCNEC immunohistochemistry staining are defined by positivity for neuroendocrine markers such as chromogranin A, synaptophysin, and CD56 (1). Neuroendocrine cells typically lack PSA and PSAP expression that is identified in adenocarcinoma, however some case reports have identified PSA staining in this setting. LCNEC can be differentiated from small cell histology by their respective morphologic features. LCNEC is composed of large island sheets of amphophilic cells with large nuclei, coarse chromatin, and prominent nucleoli (1). In contrast, small cell carcinoma is classically described as a high-grade tumor with a lack of prominent nucleoli, nuclear molding, fragility, and crush artifacts (1). Both small cell and large cell neuroendocrine carcinoma of the prostate have been shown to have worse outcomes than classical adenocarcinoma of the prostate. Patients with rapidly progressing prostate cancer or progression of disease in the setting of low or modestly rising PSA should be considered for the presence of a neuroendocrine tumor (2).

Like many other prostatic malignancies, LCNEC diagnosis is heavily dependent on the pathology of the tumor, with core needle biopsy being the preferred sample. Under microscopic examination, LCNEC is described as a high-grade feature (>10 mitotic figures in 2 mm2 of viable tumor) showing neuroendocrine differentiation (3). Unlike its counterparts of small cell tumors, its morphology is highlighted by peripheral palisades and large nests of cells with necrosis, with cells much larger than small cell and prostate adenocarcinoma. These large nests or sheets of tumor cells stain positive with at least one neuroendocrine marker, some of which include chromogranin A or synaptophysin (4). While type I LCNEC often has gene expression including TP53, KEAP1, STK11, type II LCNECs paradoxically has biallelic inactivation of TP53 and RB-1 and decreased gene expression of neuroendocrine markers, ASCL1 low/DLL3 low/NOTCH high (5).

Due to the rarity of LCNEC, the complete clinicopathological course, prognosis, and treatment have yet to be fully documented or standardized. Most of the documented information in the literature appears as either single case reports or a small case series; very few case reports mention treatments and patient outcomes. Of the cases reported in the literature, LCNEC can be divided into two main subsets: *de novo* LCNEC present at diagnosis or progression of previously diagnosed prostate adenocarcinoma (6). While systematic reviews have been completed on specific features and outcomes of LCNEC, trials of therapies and management have not yet been reviewed. Due to the aggressive nature and often poor prognosis of LCNEC tumors, it is necessary to consolidate these complex cases and various therapies available with associated outcomes. In this study, we systematically reviewed various treatment regimens and outcomes of treatment in cases of patients with LCNEC.

## Materials and methods

A systematic search was conducted in April 2022 through PubMed, Embase, and Cochrane using the following search terms: “large cell prostate cancer” and “neuroendocrine prostate”. The inclusion criteria were large cell neuroendocrine prostate cancer or mixed pathology with components of large cell neuroendocrine tumor cases with their treatment discussion. The exclusion criteria included cases not translatable to English and cases without treatment discussion. Articles that met the criteria were thoroughly reviewed and relevant data was extracted. Cases were subcategorized further into 2 subtypes depending on tumor origin: (1) primary prostate adenocarcinoma treated with long-term ADT transforming to LCNEC and (2) *de novo* LCNEC of the prostate (with no history of ADT or prior primary prostate tumor). The relevant clinical and pathological course of these tumors and treatment options and outcomes were summarized. 12 patients were found in the transformation group. 13 patients were found in the *de novo* group including one additional case treated at our institution.

## Results

### Search results and patient characteristics

**Figure 1:**
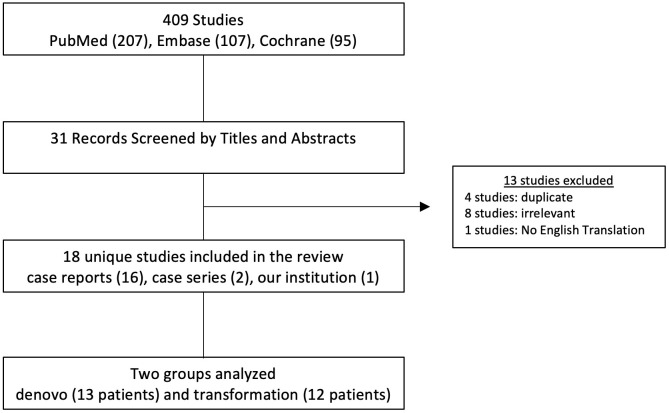
Methods for conducting a systematic review. Flowchart demonstrating the search criteria for case reports and case series concerning large cell neuroendocrine carcinoma of the prostate. Cases were divided into denovo and transformation cohorts. Exclusion criteria has been included. One case study was from our home institution.

A total of 409 studies were identified during the systematic literature search. After screening titles and abstracts and removing duplicates, 31 studies were selected. 18 studies met the inclusion criteria after reviewing the full manuscript (**Figure 1**). A total of 25 patients with a mean age of 70.4 (range 43 - 87) were included in the review. Of these, 13 patients were diagnosed with *de novo* LCNEC of the prostate, and the other 12 patients were transformation cases with a history of prostatic adenocarcinoma treated with hormonal therapy.

### *De novo* LCNEC of the prostate results

A review of the literature revealed 12 reported cases of *de novo* LCNEC of the prostate (**Table 1**). Two cases were excluded from the systematic review. The first case was excluded due to concurrent bladder cancer complicating analysis. The second case was excluded due to minimally reported outcomes. An additional unpublished *de novo* case from our institution was added to a total of 13 cases that were reviewed. At the time of diagnosis, the mean age was 68.6 (range 48-87). The presenting symptoms were consistently common lower urinary tract symptoms such as dysuria, nocturia, urinary retention, and obstructions. In the original biopsy of the prostate, the Gleason scores ranged from 7 to 9 in 5 of the 8 reported. The serum PSA values at the initial diagnosis of prostatic LCNEC were reported in 12 patients with a mean of 24.6 ng/ml (range: 0.09-170 ng/ml). 12 out of 13 cases had local spread or distant metastatic disease at the time of diagnosis. Local invasion of the bladder, pelvic wall, and pelvic lymph nodes was common. The most common areas of distant metastases were retroperitoneal, iliac, mediastinal, and para-aortic lymph nodes, colon, and bone. Three patients had eventual brain involvement with leptomeningeal disease or cerebellar and parietal lobe disease.

**Table 1:** Patient characteristics of De Novo LCNEC of the prostate.

Author, year	Age	Gleason score	PSA at diagnosis	Metastatic site at diagnosis	Initial treatment	Outcomes
Okoye et al., (7)	48	3 + 2 = 5	-	Pelvic lymph nodes	Neoadjuvant- taxol, VP16, cisplatin prostatectomy. Adjuvant Lupron	Died after having leptomeningeal disease at 1 year
Evans et al., (8)	69	3 + 2 = 5	4.3		Radical Prostatectomy Carboplatin, VP-16	Died of disease, less than 12 months; pelvic mass post RP and brain mets
Sleiman et al., (11)	68	3 + 4 = 7	6.67 with repeat of 9.65	Bladder neck and trigone	Radical prostatectomy, partial cystectomy. Adjuvant cisplatin/etoposide, radiotherapy. Triptorelin added after rising PSA	Progression-free survival of 54 months
Tzou et al., (12)	66	unavailable	2.44	Intestinal, ureteral, pelvic wall	Cisplatin/etoposide. Patient refused cystoprostatectomy	Advanced prostatic carcinoma with intestinal, ureters, and pelvic wall invasion at 3 months.
Basatac et al., (13)	70	unavailable	3.9	Bladder neck and trigone, rectosigmoid colon, iliac and paraaortic lymph nodes, bone, cerebellum, and parietal lobe	Channel TURP Adjuvant cisplatin/etoposide	Died at 7 months after Rapid progression
Acar et al., (9)	70	unavailable	<0.2	Cervical, mediastinal, abdominal, and pelvic lymph nodes, bone mets	177Lu DOTATATE	Had new bone mets at 11 months with an increase of PSA from 0.2 to 5.4
Acosta-Gonzalez et al., (10)	66	unavailable	48	Bladder neck and trigone, pelvic floor, retroperitoneal and pelvic LN	TURP	unavailable
Miyakawa et al., (14)	87	2 + 3 = 5	3.3	Bladder	Radical cystoprostatectomy adjuvant ADT	Progression-free survival at 40 months
Azad et al., (15)	70	4 + 5 = 9	9.6	Retroperitoneal lymph nodes	goserelin and bicalutamide	Progression-free survival at 15 months
Azad et al., (15)	71	4 + 5 = 9	170	retroperitoneal and iliac lymph nodes, bone	goserelin and bicalutamide	Progression-free survival at 30 months
Zafarghandi et al., (16)	71	unavailable	0.09	large pelvic mass with pelvic lymphadenopathy	Palliative radiotherapy	Not reported
Fukagawa et al., (17)	83	4 + 5 = 9	22.47	bladder, para-aortic lymph nodes, bones, bilateral lungs	cisplatin, etoposide, LHRH antagonist	Chemo terminated after 10 cycles due to myelosuppression and started abiraterone. No evidence of disease progression 20 months after diagnosis.
The case at our institution	53	4 + 3 = 7	25	Bladder neck, Ileum, ureter, mediastinal LN	Cisplatin/etoposide and Leuprorelin, pembrolizumab with cycle 2. Docetaxel was added due to progression.	The patient is currently still living at 16 months. The most recent PSA value is 0.01 ng/mL

Pathology review showed sheets, nests, and trabeculae of mitotically active large hyperchromatic cells with prominent nuclei with stippled chromatin that are consistent with large cell neuroendocrine disease. Eight cases noted high mitotic activity or Ki67 of greater than 50%, while only one noted a Ki67 of less than 10%. Seven cases reported the presence of concurrent acinar adenocarcinoma within the specimen. Immunohistochemical stains that were consistently positive were synaptophysin, chromogranin A, and Ki-67. Stains for PSA, PAF, TTF-1 were inconsistent. The mean overall survival for all *De Novo* cases was 21.5 months (range 7-54 months). The three cases that received only cisplatin/etoposide-based chemotherapy had poor survival of 7.3 months (range 3-12 months) (5, 7, 8). It is noted that these three patients who received chemotherapy alone had pure LNEC pathology with no adenocarcinoma components. They all presented at an advanced stage of the disease with heavy disease burden and two with visceral crisis (intestine, colon, and brain metastasis) on presentation. In addition, the patient in the first case also had pure LCNEC pathology and was treated with a combination of chemotherapy with ADT (4). This patient had a poor survival outcome with 13 months of survival after his radical prostatectomy and chemotherapy initiation. On the other hand, the mean survival among the three patients treated with ADT alone was 28.3 months, median was 30 months (9, 10). These three patients were noted to have mixed LCNEC and adenocarcinoma pathology. Three patients received both chemotherapy and ADT with an average survival outcome of 27.3 months and median survival of 20 months, and one case that received chemotherapy and ADT was switched to abiraterone due to myelosuppression with no evidence of progression at 20 months. These four cases also presented with mixed LCNEC and adenocarcinoma pathology. It was noted that patients with pure LCNEC pathology tend to present at a later stage with worse survival outcomes than those with mixed pathology. Of these treatment groups, 5 patients also received surgery with transurethral resection of the prostate (TURP) or radical prostatectomy in addition to the systematic treatment in each group. One patient was treated with TURP alone, however the results were not reported. Finally, one patient treated with DOTATATE had progression with bone metastasis in 11 months.

Regarding the case at our institution, this is a 53-year-old male with a 30-pack-year smoking history who initially presented to the emergency department with urinary obstruction. Further work-up revealed LCNEC of the prostate affects the bladder neck. Initial treatment with cisplatin/etoposide and later the addition of pembrolizumab and Leuprolide was unsuccessful in preventing the tumor growth. This was followed by docetaxel-based therapy (75mg/m2) with prednisone. After 5 cycles, imaging showed improvement of the sclerotic lesions and no new metastatic disease with PSA value remaining < 0.01 ng/mL for 15 months to date. This case report demonstrates that docetaxel and prednisone can be utilized as an effective therapy for patients with LCNEC of the prostate.

### LCNEC of the prostate transformation results

A review of the literature revealed 12 cases of LCNEC of the prostate that arose from a previous conventional prostatic adenocarcinoma that was initially treated with ADT. At the time of diagnosis for LCNEC, the mean age was 69.08 years (range 43-81). All patients were initially diagnosed with conventional prostatic adenocarcinoma with further differentiation into LCNEC. Metastatic LCNEC from another primary site was excluded clinically in all cases. In the original biopsy of prostatic adenocarcinoma, the Gleason scores ranged from 6 (3 + 3) to 10 (5 + 5) for the 11 cases available (**Table 2**). The average serum PSA values at the initial diagnosis of prostatic adenocarcinoma were found in 10 patients to be 28.05 ng/ml (range: 0-90 ng/ml, median 23.2 ng/ml). The initial treatment for prostatic adenocarcinoma included primary ADT (9), radical prostatectomy (2), bilateral orchiectomy (1), and palliative surgery for metastatic lesions (1). The primary regimen of ADT was bicalutamide/enzalutamide and a GnRH agonist (triptorelin). The mean duration of ADT treatment was 3.5 years (range 2-9 years). The mean interval between initial diagnosis of prostate adenocarcinoma and LCNEC diagnosis was 4.7 years total (range 2-9).

**Table 2:** Patient characteristics of post-ADT LCNEC of the prostate.

Author, year	Age	Gleason score, PSA at initial diagnosis	PSA at transformation	Metastatic sites at diagnosis	Initial treatment	Interval to LCNEC	Treatment	Outcomes
Saito et al., (18)	53	GS 4 + 5 PSA	unavailable	Scapula and bilateral iliac bone	robotic prostatectomy and retroperitoneal lymph node dissection; Triptorelin and bicalutamide for continuous elevation in PSA; palliative docetaxel and prednisone for metastatic.	3 years	palliative carboplatin and etoposide then transitioned to M6620 (ART inhibitor) and cisplatin	20 months of non-CNS progression-free survival
Aljarba et al., (19)	79	GS 4 + 5 PSA	12.3	Brain metastasis	Surgical removal of right frontal lobe lesion	9 years	none	Neurological status declined after brain met resection. Cardiac arrest 43 days after surgery.
Evans et al., 2006 (8)	71	GS 10/10 PSA unavailable	3.66	unavailable	ADT for 3 years	3 years	Palliative mitoxantrone	Died of disease with bone, lymph nodes mets
Evans et al., (8)	81	GS 7/10 PSA 10.5	<0.05	unavailable	Watchful waiting for 3 years, then ADT for 2 years	12 years	Carboplatin, etoposide	Died of disease with lung, liver, brain mets
Evans et al., (8)	75	Unavailable	unavailable	unavailable	Radical prostatectomy then ADT 9 years post prostatectomy	12 years	Palliative radiation	Lost to follow up
Evans et al., (8)	64	GS 7/10 PSA 23.2	<0.3	unavailable	Bilateral orchiectomy followed by ADT	4 years	Carboplatin, etoposide	Died of disease with bone mets
Evans et al., (8)	65	GS 8/10 PSA 29.7	<0.2	unavailable	ADT for 2 years	2 years	Carboplatin, etoposide	Died of disease with bone mets
Evans et al., 2006 (8)	43	GS 8/10 PSA 68	9.9	unavailable	ADT for 2 years	2 years	Carboplatin, etoposide, mitoxantrone	Died of disease with bone mets
Patel et al., (20)	75	GS 5 + 4, PSA 7.5	0.1	unavailable	ADT for 2 years	2 years	Palliative radiation	Unclear outcomes
Schepers et al., (21)	75	GS unavailable PSA 90	unavailable	para-aortal LAD, bone mets	unavailable	1.5 year	none	Died a few weeks after diagnosis
Wynn et al., (22)	73	GS unavailable PSA 9	unavailable	sigmoid colon, omentum, peritoneum	External beam radiation	9 years	None	Died three weeks after diagnosis
Charcos et al., (23)	81	GS and PSA unavailable	0	liver, lung, pleura; mediastinal, diaphragmatic, retroperitoneal and iliac LAD	ADT for 5 years	5 years	Docetaxel and carboplatin	Died ten days after chemotherapy initiation

Serum PSA at the time of LCNEC diagnosis was undetectable in three patients, and in all cases, was much lower than the initial PSA level at the time of prostate adenocarcinoma diagnosis (**Table 2**). The mean PSA value at the time of LCNEC diagnosis was 3.3 ng/ml (range: 0-9.3 ng/ml). The pattern of metastasis of LCNEC resembles the pattern of metastasis of prostate adenocarcinoma with common sites of metastasis including pelvic side wall, bone, lung, liver, and sigmoid colon. There were three transformation cases with rare brain metastasis.

Common immunohistochemical staining patterns for LCNEC of the prostate included chromogranin A, synaptophysin, CD56/NCAM, and CD57/Leu7. These cancers were typically negative for PSA, PSAP, and androgen receptors. Metastasis commonly showed somatostatin receptor positivity. After the diagnosis of LCNEC of the prostate, patients were treated with carboplatin, etoposide (7), carboplatin, etoposide, palliative mitoxantrone (1), palliative mitoxantrone (1), radiation (1), and palliative care. Patients who received platinum-based chemotherapy (cisplatin/carboplatin) with a DNA topoisomerase II inhibitor (etoposide) had a mean survival of 7 months (5 patients). There is one clinical case that reported an impressive progressive free survival (PFS) after diagnosis of LCNEC of 20 months. This case was being treated with cisplatin/etoposide-based therapy in conjunction with a novel ATR inhibitor (M6620).

## Discussion

LCNEC can occur by neuroendocrine transformation. One potential mechanism was described by Cerasuolo et al. called trans-differentiation. This *in-vitro* study was completed on androgen-sensitive human prostate adenocarcinoma (LNCaP) cells. When LNCaP were cultured in hormone-deprived conditions, they have the potential to transdifferentiate to a neuroendocrine-like cell (24). From this, in-silico models then predicted these neuroendocrine-like cells could support the survival of other LNCaP cells leading to the development of a hormone refractory state (24). This mechanism could be the initial steps of neuroendocrine transformation in prostate adenocarcinoma treated with ADT. While serum PSA level is a reliable tumor marker for prostate adenocarcinoma, serum PSA levels proved to be an unreliable marker for neuroendocrine prostate cancer. In our review, 11 out of 25 patients had normal or undetectable PSA at the time of LCNEC diagnosis. The mean PSA level during the transformation was low at 4.2 ng/ml, contrasting with the higher 28.05 ng/ml at the initial diagnosis of prostatic adenocarcinoma. Therefore, diagnosing LCNEC remains challenging, emphasizing the necessity of relying on tissue biopsy for accurate identification due to the limitations of PSA levels in reflecting neuroendocrine transformations in the prostate.

Prostate cancer, in general, has been known to spread most commonly to the paraaortic and pelvic lymph nodes. Lymph node metastasis has been demonstrated to be positively associated with hematogenous spread. Common sites of hematogenous metastasis from most common to least include bone, lung, liver, pleura, and adrenals (25). Both *de novo* and transformation cases in this review follow the same pattern of metastasis. The most common areas of metastasis include bone (13), local regional lymph node (10), bladder/upper urinary tract (8),brain (5), retroperitoneal lymph node (4), lung (5), colon (3), omentum and peritoneum (1). Notably, there are significantly higher rates of brain and visceral crises in LCNEC cases. Six out of the 23 reported cases demonstrated metastasis to the brain, indicating a notably elevated incidence of brain metastasis in comparison to prostate adenocarcinoma. This underscores the distinctive metastatic behavior of LCNEC, emphasizing the importance of comprehensive monitoring and targeted management strategies for patients with this aggressive form of prostate cancer.

Paraneoplastic syndromes in large cell prostate cancer are exemplified by three reported cases. Schepers et al. reported a patient with LCNEC diagnosed with Cushings syndrome, characterized by the exogenous secretion of adrenocorticotropic hormone (ACTH). This individual underwent treatment with spironolactone and ketoconazole, resulting in the subsequent control of Cushings syndrome (21). This individual underwent treatment with spironolactone and ketoconazole, resulting in the subsequent control of Cushings syndrome. In a second case reported by Papagoras et al, the patient manifested with dermatomyositis and concurrent polycythemia (26). Due to the co-occurrence of these two conditions, a malignancy workup was initiated and revealed a metastatic prostate cancer that was confirmed on biopsy to be LCNEC of the prostate. Interestingly, this patient did not endorse any urinary symptoms and his elevated PSA of 11.49 was not detected until his malignancy workup. A third case was reported that demonstrated a patient presenting initially with central diabetes insipidus which later revealed LCNEC with parietal and cerebellar metastases (13). The patient initially improved with desmopressin and lyophilisate, however, he subsequently developed nephrogenic DI related to dexamethasone administration that was not responsive to desmopressin. Notably, large cell prostate cancer is associated with distinct paraneoplastic syndromes, including hypercalcemia, Lambert-Eaton syndrome, Cushings syndrome, and syndrome of inappropriate antidiuretic hormone secretion (SIADH). These diverse paraneoplastic manifestations underscore the complexity of large cell prostate cancer and emphasize the need for comprehensive clinical management strategies to address associated syndromes effectively.

Large cell neuroendocrine carcinoma (LCNEC) is characterized by an aggressive clinical course and an unfavorable prognosis. Most of the cases of *de novo* LCNEC presented with metastasis and advanced disease at diagnosis. This may suggest that there is a faster progression with high mitotic activity in cases of *de novo* LCNEC compared to adenocarcinoma. This is also supported by the fact that the Ki67 is >50% in the majority of cases, with only one case having a Ki67 of <10%. It was also noted that patients with pure LCNEC pathology tend to present at a later stage with worse survival outcomes than those with mixed pathology. The low PSA values at the time of transformation pose challenges for clinicians to detect transformation once it occurs. Many transformation LCNEC cases were detected at an advanced stage where the cancer already spread to distant organs. Therefore, we cannot solely rely on PSA values to monitor tumor recurrence or LCNEC transformation. Similarly, in the *De novo* population, the advanced disease at diagnosis could be because PSA levels may be less reliably expressed in LCNEC compared to adenocarcinoma. Only about half of the cases had elevated PSA at the time of diagnosis, or moderately elevated PSAs despite the extent of metastasis. Around 1% of primary prostate cancer and 25-30% of metastatic castrate-resistant prostate cancer further differentiate into a neuroendocrine phenotype (1). The current screening guidelines solely rely on PSA values to monitor tumor recurrence which makes it difficult to detect neuroendocrine prostate tumors as most of them present with low or undetectable PSA. It would be ideal to be able to detect these highly aggressive neuroendocrine tumors at an earlier stage but implementing screening guidelines for neuroendocrine markers is low diagnostic yield and may lead to unnecessary testing and pose a cost burden to patients with prostate cancer.

The optimal treatment for LCNEC of the prostate remains unclear. Due to the rarity of the disease, the treatment for LCNEC of the prostate has been extrapolated from that for large cell lung cancer and prostate adenocarcinoma. In many cases, patients were treated with either platinum-containing chemotherapy including a combination of carboplatin or cisplatin with etoposide or docetaxel, androgen deprivation therapy, or a combination of the two treatments (27). Many *de novo* case reports continued to use ADT as the backbone of therapy with encouraging results (mean survival of 28.3 months and median of 30 months with ADT alone and mean survival of 27.3 months and median of 16 months with both ADT and chemotherapy vs 7.3 months for chemotherapy alone). There appears to be a benefit to adding ADT despite inconsistent PSA staining on IHC for the *de novo* group, however, whether this can be generalized to all *de novo* patients is a more complicated discussion due to the low sample size (N=3 and possibly more severe underlying disease. The cases treated with chemotherapy alone were all pure LCNEC and were diagnosed and higher stages. The lack of traditional prostate adenocarcinoma on the histology may have factored into the decision to forgo ADT. The single case with pure *de novo* LCNEC that was treated with both chemo and ADT did have a slightly longer PFS of 13 mo, however, this is still much shorter than the PFS seen in mixed histologies. What is clear, however, is that patients with a mixed phenotype with LCNEC and adenocarcinoma pathology appear to benefit from receiving ADT, while pure LCNEC had worse survival than mixed histology.

In our study, the mean documented survival of those patients in the transformation group who were treated with conventional chemotherapy including platinum-based therapy was 148 days (4.9 months) (ranging from 10 days to 7 months). Similarly, among the *de novo* group the patients treated with chemotherapy alone without ADT had poor overall survival of 5 months. Several cases showed that the patients progressed quickly despite initial PSA response to chemotherapy with overall poor outcomes. The classic definite treatment options such as surgery or radiation were usually not able to be implemented, except in the one case where a diagnosis was made before metastasis. Additionally, due to fast progression and poor survival outcomes, only in 2 of the cases were multiple lines of therapy able to be implemented. This finding is consistent with a previous systematic review in which nearly all patients who had transformation cases died quickly after the diagnosis of LCNEC irrespective of treatment types (27). In a systematic review of neuroendocrine prostate cancer, survival of patients following first-line treatment with platinum and etoposide regimen is poor with a median survival of 7 months (28). Therefore, although ADT still appears to have a role in the treatment of *de novo* LCNEC, there is an unmet need to develop more effective treatments for LCNEC that will allow for more lines of therapy to be implemented.

Of note, one patient in the transformation case with a somatic BRCA2 mutation was treated with a combination of M6620 and chemotherapy including gemcitabine, cisplatin, and etoposide with an impressive PFS of 20 months (18). M6620 is an emerging, potent ataxia telangiectasia mutated and Rad3-related protein (ATR) inhibitor. ATR plays an important role in the DNA damage response (DDR) pathway, and inhibition of ATR may lead to synthetic lethality in tumors dependent on intact ATR function (29). This is so far the first case that reports a long and durable response to the combination of M6620 and cisplatin in a patient with metastatic LCNEC. The case suggests that chemotherapy alone may not be adequate in controlling high-grade neuroendocrine prostate cancer, and a combination of platinum therapy and ATR inhibitor should be considered especially in patients with mutations in genes involved in homologous recombination repair pathway. In addition, the patients case at our institution also had a good survival outcome of 15 months to date. Noted that the patient did not respond to the initial treatment of cisplatin/etoposide, pembrolizumab, and Lupron. This case may suggest that docetaxel and prednisone can be utilized as an effective therapy for patients with LCNEC of the prostate.

## Conclusion

LCNEC of the prostate is a rare disease with an aggressive clinical course and often a poor prognosis. Diagnosing LCNEC could be challenging since the PSA level at the time of transformation is normally undetectable or low. Most of the LCNEC cases in this review are detected at the time of advanced stage with metastasis. The survival outcome was poor (average of 5 months) in patients presenting with distant metastasis who received conventional platinum-based chemotherapy with etoposide. Although ADT still appears to have a role in the treatment of *de novo* LCNEC, there is an unmet need to develop more effective treatments for LCNEC.

## Data availability statement

The original contributions presented in the study are included in the article/supplementary material. Further inquiries can be directed to the corresponding author.

## Author contributions

NN: Conceptualization, Data curation, Formal analysis, Investigation, Methodology, Project administration, Validation, Writing – original draft, Writing – review & editing. RF: Conceptualization, Data curation, Formal analysis, Investigation, Methodology, Validation, Writing – original draft, Writing – review & editing. OM: Conceptualization, Data curation, Formal analysis, Investigation, Methodology, Software, Supervision, Validation, Writing – original draft, Writing – review & editing. RH: Conceptualization, Data curation, Formal analysis, Investigation, Methodology, Software, Supervision, Validation, Writing – original draft, Writing – review & editing. CS: Conceptualization, Data curation, Formal analysis, Investigation, Methodology, Software, Supervision, Validation, Writing – original draft, Writing – review & editing. RK: Conceptualization, Data curation, Formal analysis, Investigation, Methodology, Software, Supervision, Validation, Writing – original draft, Writing – review & editing. BA: Conceptualization, Data curation, Formal analysis, Funding acquisition, Investigation, Methodology, Project administration, Resources, Supervision, Validation, Visualization, Writing – original draft, Writing – review & editing.
